# Allied Health Professional Support in Pediatric Inflammatory Bowel Disease: A Survey from the Canadian Children Inflammatory Bowel Disease Network—A Joint Partnership of CIHR and the CH.I.L.D. Foundation

**DOI:** 10.1155/2017/3676474

**Published:** 2017-05-16

**Authors:** Wael El-Matary, Eric I. Benchimol, David Mack, Hien Q. Huynh, Jeff Critch, Anthony Otley, Colette Deslandres, Kevan Jacobson, Jennifer deBruyn, Matthew W. Carroll, Eytan Wine, Johan Van Limbergen, Mary Sherlock, Kevin Bax, Sally Lawrence, Ernest Seidman, Robert Issenman, Thomas D. Walters, Peter Church, Anne M. Griffiths

**Affiliations:** ^1^Section of Pediatric Gastroenterology, Winnipeg Children's Hospital, Max Rady College of Medicine, Rady Faculty of Health Sciences and Children's Hospital Research Institute, Winnipeg, MB, Canada; ^2^Department of Pediatrics, University of Alexandria, Alexandria, Egypt; ^3^Department of Pediatrics, University of Ottawa and Children's Hospital of Eastern Ontario, Ottawa, ON, Canada; ^4^School of Epidemiology, Public Health and Preventive Medicine, University of Ottawa, Ottawa, ON, Canada; ^5^Edmonton Pediatric IBD Clinic (EPIC), Department of Pediatrics, University of Alberta, Edmonton, AB, Canada; ^6^Department of Pediatrics, Janeway Children's Hospital, St. John's, NL, Canada; ^7^Division of Pediatric Gastroenterology & Nutrition, Department of Pediatrics, IWK Health Centre, Dalhousie University, Halifax, NS, Canada; ^8^Department of Pediatrics, CHU Sainte-Justine, Montréal, QC, Canada; ^9^Division of Pediatric Gastroenterology, Hepatology and Nutrition, Department of Pediatrics, BC Children's Hospital, UBC, Vancouver, BC, Canada; ^10^Section of Pediatric Gastroenterology, Department of Pediatrics, University of Calgary, Calgary, AB, Canada; ^11^Division of Pediatric Gastroenterology, McMaster Children's Hospital, Hamilton, ON, Canada; ^12^Department of Pediatrics, Schulich School of Medicine, Western University, Children's Hospital of Western Ontario, London, ON, Canada; ^13^Division of Pediatric Gastroenterology, Hepatology and Nutrition, Montreal Children's Hospital, Department of Pediatrics, McGill University, Montreal, QC, Canada; ^14^Division of Pediatric Gastroenterology, Hepatology and Nutrition, Hospital for Sick Children, Department of Pediatrics, University of Toronto, Toronto, ON, Canada

## Abstract

**Objectives:**

The current number of healthcare providers (HCP) caring for children with inflammatory bowel disease (IBD) across Canadian tertiary-care centres is underinvestigated. The aim of this survey was to assess the number of healthcare providers (HCP) in ambulatory pediatric IBD care across Canadian tertiary-care centres.

**Methods:**

Using a self-administered questionnaire, we examined available resources in academic pediatric centres within the Canadian Children IBD Network. The survey evaluated the number of HCP providing ambulatory care for children with IBD.

**Results:**

All 12 tertiary pediatric gastroenterology centres participating in the network responded. Median full-time equivalent (FTE) of allied health professionals providing IBD care at each site was 1.0 (interquartile range (IQR) 0.6–1.0) nurse, 0.5 (IQR 0.2–0.8) dietitian, 0.3 (IQR 0.2–0.8) social worker, and 0.1 (IQR 0.02–0.3) clinical psychologists. The ratio of IBD patients to IBD physicians was 114 : 1 (range 31 : 1–537 : 1), patients to nurses/physician assistants 324 : 1 (range 150 : 1–900 : 1), dieticians 670 : 1 (range 250 : 1–4500 : 1), social workers 1558 : 1 (range 250 : 1–16000 : 1), and clinical psychologists 2910 : 1 (range 626 : 1–3200 : 1).

**Conclusions:**

There was a wide variation in HCP support among Canadian centres. Future work will examine variation in care including patients' outcomes and satisfaction across Canadian centres.

## 1. Introduction

The inflammatory bowel diseases (IBD) and the main subtypes Crohn's disease (CD) and ulcerative colitis (UC) have a considerable impact on patients' health, social functioning, and quality of life that may result in significant demand on healthcare resources. In 2012, the burden-of-illness report from Crohn's and Colitis Canada estimated that the direct medical costs of IBD in Canada were over one billion dollars, primarily funded through the Canadian public healthcare system [1]. The report also highlighted the presence of a considerable gap between the perceived ideal and actual IBD care.

Quality improvement (QI) in medicine is defined as the utilisation of an evidence based approach in order to make meaningful, positive changes to healthcare [2, 3]. Clinical quality indicators are quantitative endpoints used to guide, monitor, and improve the quality of patients' care [2]. The use of QI in IBD is becoming increasingly recognized as a methodology for improving IBD care [3]. It is important to understand that these QI measures are not designed to reflect ideal care, but rather the minimum expected standard of care based on the best available evidence [4].

There is significant variability in the delivery of medical care to persons with IBD [5]. The “IBD Standards Group” of the UK indicated that, for effective IBD clinical care, a multidisciplinary approach with allied healthcare professional support is imperative. Allied healthcare professional support includes but is not limited to IBD nurses, dietitians, clinical psychologists, and social workers [6]. A multidisciplinary IBD team with several allied healthcare professional members such as nurses, dietitians, clinical psychologists, and social workers may provide significant support towards a more accurate assessment of several quality outcome indicators. There is paucity in the pediatric literature on definitions of optimal standards of care in children with IBD. Moreover, data are lacking on how the outcome of care would vary with implementation of different standards of care and involvement of the various allied healthcare personnel.

We recently conducted a survey among pediatric IBD centres in Canada as a part of a quality improvement (QI) initiative to collect data on the availability of allied healthcare professional support. The aim of the survey was to assess current allied healthcare professional support in the provision of care to children with IBD in pediatric tertiary-care centres across Canada.

## 2. Material and Methods

The Canadian Children Inflammatory Bowel Disease Network: A Joint Partnership of CIHR and the CH.I.L.D. Foundation is a network established in 2014. This network comprises 12 pediatric IBD tertiary-care centres across Canada and is tasked with the creation of an inception cohort of all children diagnosed with IBD. The network also aims to standardize and optimize clinical practice across Canada and to identify quality of care indicators to further improve outcomes for Canadian children with IBD.

A self-administered questionnaire was designed to examine the total number of current IBD patients and available resources for each centre; this included the number of full-time equivalent (FTE) physicians, nurses/physician assistants, dietitians, social workers, and clinical psychologists/child life worker caring for children with IBD. Additional questions also addressed wait times for a new probable IBD referral to be seen by a pediatric gastroenterologist, to undergo diagnostic endoscopy, colonoscopy, and magnetic resonance enterography (MRE) after their first meeting with the pediatric gastroenterologists and to receive pathology reports for biopsy sample analysis. The full questionnaire is available in Supplementary Material available online at https://doi.org/10.1155/2017/3676474.

The questionnaire was initially piloted among a small group of gastroenterologists and refined before being distributed via email to all clinical members of the Canadian Children IBD Network. Validation of answers was established by sending the questionnaire to more than one member from the same centre (when available) and asking them to answer questions independently. Moreover, the survey was accompanied with clear instructions on how to respond to each question and the availability of the first author (WEM) to clarify any possible confusion around any of the questions. When there was a difference, the mean of the numbers provided was taken as the number representative of the centre. The survey asked respondents to provide the total number of full-time equivalent (FTE) clinical gastroenterologists in each centre in addition to total number of FTE clinical gastroenterologists who provide care mainly to children with IBD. Centres were classified as follows:

- Centres with dedicated IBD pediatric gastroenterologists (defined as the majority of their clinical workload dedicated to IBD patient care)
- Centres where pediatric gastroenterologists were providing care to children with all gastrointestinal problems, including IBD

Median numbers with interquartile range (IQR) of healthcare providers (HCP) (physicians and allied health workers) across Canada were calculated. Ratios of HCP to IBD patients in each centre were also calculated and compared using the Pearson Chi square test.

Calculations and data analysis were performed using SPSS (Statistical Package for the Social Sciences, IBM Corp. 2013, IBM SPSS Statistics for Windows, version 22.0. Armonk, NY: USA). In all analyses, *p* < 0.05 were considered statistically significant.

## 3. Results

**Figure 1:**
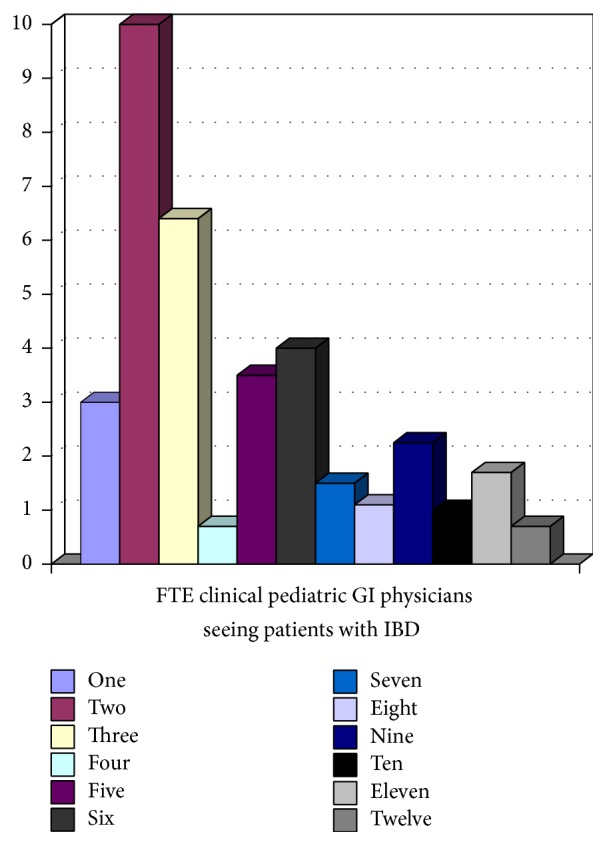
Full-time equivalent (FTE) pediatric gastroenterologists for children with IBD per centre.

**Figure 2:**
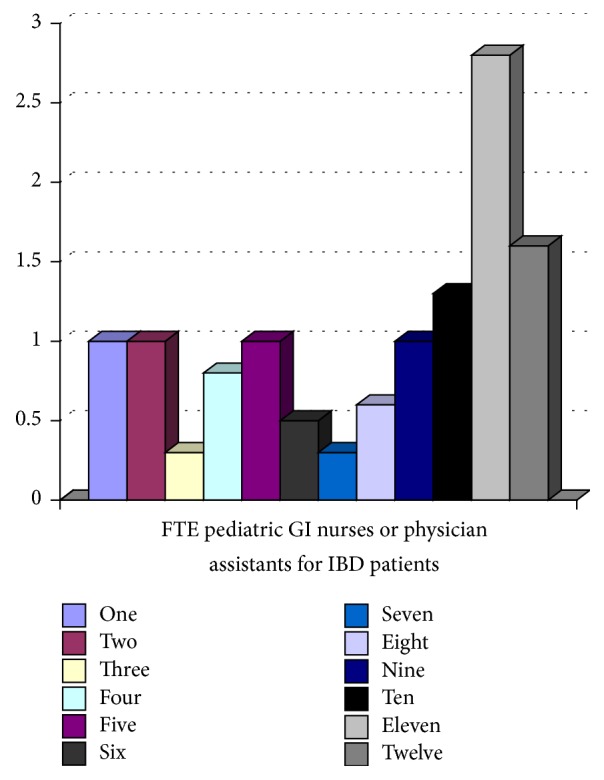
Full-time equivalent (FTE) nurses for children with IBD per centre.

**Figure 3:**
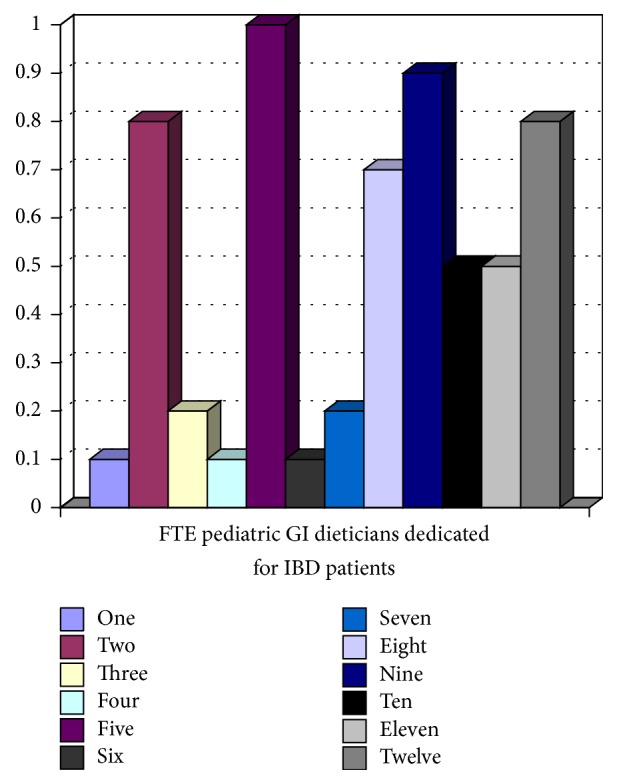
Full-time equivalent (FTE) dietitians for children with IBD per centre.

**Figure 4:**
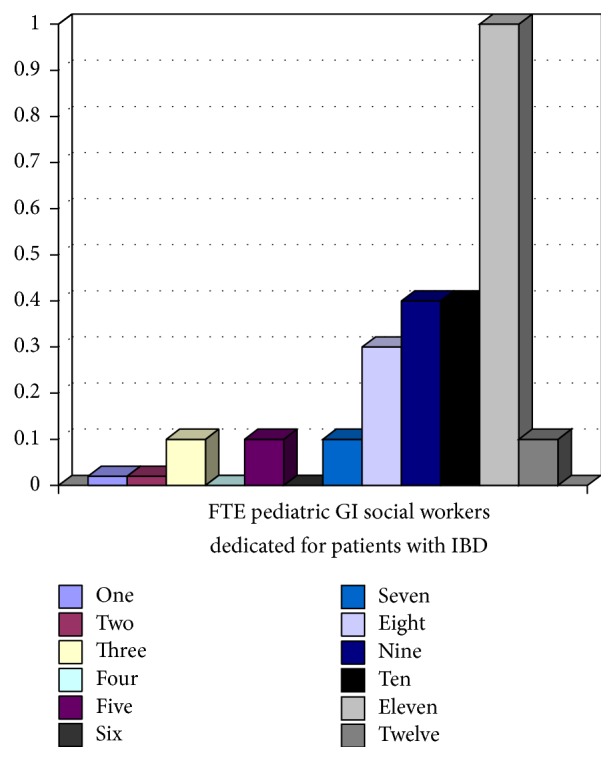
Full-time equivalent (FTE) social workers for children with IBD per centre.

**Figure 5:**
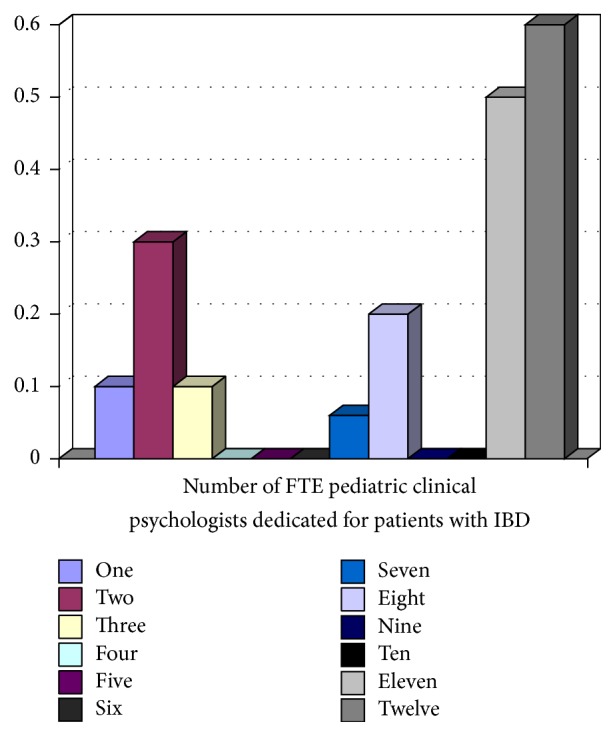
Full-time equivalent (FTE) clinical psychologist/child life workers for children with IBD per centre.

All 12 tertiary pediatric gastroenterology centres participating in the network responded. Five (41.6%) of 12 sites had dedicated IBD physicians, where IBD care was provided by specific pediatric gastroenterologists. Figures 1–5 show the number of IBD physicians, nurses, dietitians, clinical psychologists/child life workers, and social workers for each centre. Most centres do not have designated dieticians, social workers, or clinical psychologists who only see children with IBD; rather, they see those children in addition to other children with gastrointestinal and liver diseases. In addition, 2 centres (16%) did not have any social work support and 5 centres (42%) did not have a clinical psychology service to support their IBD patients (Figures 4 and 5).

The median and interquartile range (IQR) for the number of IBD physicians, nurses, dietitians, clinical psychologists/child life workers, and social workers in addition to wait time for new IBD patients to be assessed by a pediatric gastroenterologist, undergo endoscopy, receive pathology results, and undergo MRE across Canada are summarized in Tables 1 and 2. The median wait time for a consultation for a likely new IBD patient to be seen by a pediatric gastroenterologist was 2 (IQR 1–2.5) weeks, to undergo endoscopy after initial contact with pediatric gastroenterologist was 2.75 (IQR 1.5–5) weeks, and to undergo MRE after the initial consult with pediatric gastroenterologist was 9 (IQR 4–12) weeks.

Overall, the ratio of IBD patients to IBD physicians was 114 : 1 (range 31 : 1–537 : 1), IBD patients to nurses/physician assistants was 324 : 1 (range 150 : 1–900 : 1), IBD patients to dietician was 670 : 1 (range 250 : 1–4500 : 1), IBD patients to social workers was 1558 : 1 (range 250 : 1–16000 : 1), and IBD patients to clinical psychologists/child life workers was 2910 : 1 (range 626 : 1–3200 : 1).  Table 3 summarizes the ratios for HCP to IBD patients in each contributing centre. Centres across Canada had significantly different ratios of HCP to IBD patient's volume.

## 4. Discussion 

The incidence of pediatric-onset IBD is rising [7, 8], with particularly rapidly rising rates in children under 10 years old [9]. In children, active disease can have devastating consequences on growth and social functioning [10]. Watching for symptom to flare creates increased levels of anxiety and stress for both the child and the parents [11]. Consequently a multidisciplinary team with HCP including physicians, nurses, dietitians, social workers, and clinical psychologists is very important for delivering the required care for children with IBD. Evidence on the appropriate number of different IBD team members is scarce. Our survey showed that there was a wide variation in HCP support among Canadian centres. 16% of centres did not have any social work support and 42% did not have a clinical psychology service to support their IBD patients.

In 2015, the National Institute for Health and Care Excellence (NICE) of the UK published a quality standard report on persons with IBD aiming to improve attendance at school, sickness absence from work, IBD-related hospital admissions, and patients' experience of services. The report stated that healthcare services should provide an age-appropriate support from a multidisciplinary team for people with IBD and their family members or caregivers [12]. As IBD can have several impacts including physical, emotional, psychological, and social consequences, a multidisciplinary approach for IBD care has been a widely recommended strategy to reduce any possible disparities in IBD care [6, 13]. In addition to gastroenterologists and colorectal surgeons, the team should include clinical nurse specialists with interests and expertise in IBD, dietitians, psychologists, pharmacists, pathologists, and radiologists with special interests in IBD [6, 12, 14, 15].

The UK and Australian standards groups published very similar documents recommending a defined IBD team with named personnel to care for adults with IBD [6, 15]. Based on the need for cross-coverage and a defined population of 250,000, the IBD team should have a minimum of 2 FTE consultant gastroenterologists, 2 FTE consultant colorectal surgeons, 1.5 FTE clinical nurse specialists with competencies in IBD, 1.5 FTE clinical nurse specialist with competencies in stoma care and ileoanal pouch surgery, 0.5 FTE dietitian, and 0.5 FTE administrative support for IBD meetings, IBD database recording, and audits. Additionally, the team should have one named histopathologist, a radiologist, and a pharmacist with a special interest in gastroenterology. Named personnel should include specialists in psychology, rheumatology, ophthalmology, dermatology, obstetrics, and a nutrition support team with an interest in IBD [15].

A nutrition support team should be available for patients who require complex enteral and parenteral nutritional support including exclusive enteral feeding therapy for CD [6, 13, 14]. For children and adolescents with IBD, both the UK and Australian standards indicated that care should be provided by pediatric gastroenterologists with specialist nursing and dietetic support but no recommendations were provided on the size of such support [6, 13–15].

In 2013, The Canadian Digestive Health Foundation issued an important document that endorsed the UK IBD standards of care particularly with respect to the multidisciplinary approach to persons with IBD. The document added that, in some jurisdictions within Canada, the support team may also require a psychiatrist to offer advice or prescribe medications such as antidepressants. Other ancillary health providers to consider include social workers and physiotherapists. Administrative support for achieving medication coverage is also essential [16].

The European Crohn's and Colitis Organization (ECCO), in its 2013 consensus statement for improving access to nurse education in IBD, described the advanced IBD nurse as “an autonomous clinical expert in IBD who is responsible for the assessment and provision of evidence based care planning, and treatment evaluation, and who provides practical information, education and emotional support for patients with IBD” [17]. A recent audit from the Royal College of Nursing, UK, evaluated the roles, responsibilities, and services provided by IBD nurses and identified areas of improvement, concluding that IBD nurses were influencing the management of considerable numbers of patients within acute care settings. The audit recognized that, despite the increased numbers of IBD nurses, 79% of sites failed to meet the standard of 1.5 FTE IBD nurse specialists per 250,000 of population [18].

Our survey revealed a wide variability of current allied healthcare professional support across Canadian IBD tertiary-care centres. However, it is not known if children with IBD in those centres with more support have better healthcare outcomes, fewer disease complications, better long-term life events, or fewer long-term mental health issues compared to those in centres with less support. It seems evident, based on current numbers and available evidence supporting the major negative impacts of the disease on nutritional and psychosocial aspects of children with IBD, there is a need for more allied healthcare professional support in pediatric IBD in Canada especially dietitians, clinical psychologists, and social workers. Beyond the clinical needs analyses, health economic analyses need to be considered in the context of improved incremental care that may be proposed. Our next step will be examining QI outcomes including patient's satisfaction and reported outcome measures (PROMs) across different centres to assess if those with better support have improved outcomes for patients.

One limitation of the current survey was that the perception of definition of a full-time equivalent (FTE) position was likely to be variable among respondents especially when it came to FTE clinical physicians as some answers perceived one FTE as one physician regardless of the job description of that physician and the fraction of clinical versus nonclinical activities. It was also difficult to accurately estimate the exact number of IBD patients and FTE devoted to IBD patients when physicians and allied healthcare workers looked after both IBD and non-IBD patients. It is difficult to know, especially since this is a self-reported questionnaire, how the responders calculated data such as the number of patients seen or the average wait times. Nevertheless, we validated the answers through getting the answers from more than one responder per each centre and carefully examining any possible differences. Overall, the survey gave a good preliminary estimate on an area that has never been examined before. However, these estimates should be perceived in the context of those limitations.

## 5. Conclusions

As IBD has major impacts on physical, nutritional, and psychosocial aspects of children and their families, a multidisciplinary team approach with sufficient allied healthcare professional support is important to IBD care. Currently there are no evidence based recommendations to suggest the volume or the size of required healthcare professional support in pediatric IBD. This Canadian national survey provides some evidence on the current pediatric IBD workload and available support within Canada. The current support varied significantly across Canada, but the exact amount of support required by children with IBD and the association between levels of support and outcomes are uncertain. The next step will be examining outcomes including patients' satisfaction across different centres to estimate whether those centres with better support have better outcomes and then to intervene to improve the care of children diagnosed with IBD.

## Supplementary Material

The Supplementary Material displays the questions included in the self-administered questionnaire.

## Figures and Tables

**Table 1:** Median and interquartile range (IQR) of number of healthcare providers available for the care of children with IBD per pediatric centre in Canada.

Provider type	Median number of FTE	IQR
Physicians	1.98	1.1–3
Nurses/physician assistants	1	0.6–1
Dietitians	0.5	0.2–0.8
Social workers	0.3	0.2–0.8
Psychologists/child life workers	0.1	0.02–0.3

FTE: full-time equivalent.

**Table 2:** Median and interquartile range (IQR) of wait times (in weeks) for services to children with IBD in Canada.

Service type	Median (weeks)	IQR
First clinic visit	2	1–2.5
Diagnostic endoscopy	2.75	1.5–5
Pathology results	1	1–1.3
MRE	9	4–12

MRE: magnetic resonance enterography.

**Table 3:** Number of IBD patients per physicians and allied healthcare professionals in individual tertiary centres.

Center number	Number of patients per a physician	Number of patients per a nurse	Number of patients per a dietitian	Number of patients per a social worker	Number of patients per a psychologist
1	107	320	3200	16000	3200
2	50	500	625	1667	1667
3	31	667	1000	2000	2000
^*∗*^4	120	150	1200	No social worker	No psychologist
5	111	250	250	250	No psychologist
^*∗*^6	113	900	4500	No social worker	No response
7	37	184	275	2750	916
^*∗*^8	182	334	286	2000	1000
9	128	287	319	718	No psychologist
^*∗*^10	300	231	600	750	No psychologist
^*∗*^11	530	321	1800	3000	1800
12	537	235	470	3760	626
*p* value	0.004	0.000	0.010	0.04	0.003

^*∗*^Centres with dedicated IBD physicians.

## References

[1] Becker H. M., Crigat D., Ghosh S. (2015). Living with inflammatory bowel disease: a Crohn's and Colitis Canada survey. *Canadian Journal of Gastroenterology and Hepatology*.

[2] El-Matary W., Dufault B. (2016). Quality improvement in paediatric inflammatory bowel disease: the Manitoba experience. *Acta Paediatrica*.

[3] Quach P., Nguyen G. C., Benchimol E. I. (2013). Quality improvement in pediatric inflammatory bowel disease: moving forward to improve outcomes. *World Journal of Gastroenterology*.

[4] Melmed G. Y., Siegel C. A. (2013). Quality improvement in inflammatory bowel disease. *Gastroenterology and Hepatology*.

[5] Melmed G. Y., Siegel C. A., Spiegel B. M. (2013). Quality indicators for inflammatory bowel disease: development of process and outcome measures. *Inflammatory Bowel Diseases*.

[6] The IBD Standards Group (2013). *Standards for the Healthcare of People Who Have Inflammatory Bowel Disease—2013 Update*.

[7] El-Matary W., Moroz S. P., Bernstein C. N. (2014). Inflammatory bowel disease in children of Manitoba: 30 years experience of a tertiary center. *Journal of Pediatric Gastroenterology and Nutrition*.

[8] Benchimol E. I., Fortinsky K. J., Gozdyra P., Van Den Heuvel M., Van Limbergen J., Griffiths A. M. (2011). Epidemiology of pediatric inflammatory bowel disease: a systematic review of international trends. *Inflammatory Bowel Diseases*.

[9] Benchimol E. I., MacK D. R., Nguyen G. C. (2014). Incidence, outcomes, and health services burden of very early onset inflammatory bowel disease. *Gastroenterology*.

[10] Bousvaros A., Sylvester F., Kugathasan S. (2006). Challenges in pediatric inflammatory bowel disease. *Inflammatory Bowel Diseases*.

[11] Gray W. N., Graef D. M., Schuman S. S., Janicke D. M., Hommel K. A. (2013). Parenting stress in pediatric IBD: relations with child psychopathology, family functioning, and disease severity. *Journal of Developmental and Behavioral Pediatrics*.

[12] NICE quality standard: inflammatory bowel disease, NICE, 2015, https://www.nice.org.uk/guidance/QS81/chapter/Quality-statement-2-Multidisciplinary-team-support

[13] Sack C., Phan V. A., Grafton R. (2012). A chronic care model significantly decreases costs and healthcare utilisation in patients with inflammatory bowel disease. *Journal of Crohn's and Colitis*.

[14] Mowat C., Cole A., Windsor A. (2011). Guidelines for the management of inflammatory bowel disease in adults. *Gut*.

[15] Inflammatory Bowel Disease Quality of Care Program (2015). *Interim Australian IBD Standards: Standards of Healthcare for People with Inflammatory Bowel Disease in Australia—2013 Update*.

[16] Canadian Digestive Health Foundation (2013). *Best Practices in IBD Care: Taking Steps to Introduce an Integrated Multidisciplinary Patient-Centric Care Mode*.

[17] O'Connor M., Bager P., Duncan J. (2013). N-ECCO consensus statements on the European nursing roles in caring for patients with Crohn's disease or ulcerative colitis. *Journal of Crohn's and Colitis*.

[18] Inflammatory bowel disease nursing: results of an audit exploring the roles, responsibilities and activity of nurses with specialist/advanced roles, 2011, https://www2.rcn.org.uk/__data/assets/pdf_file/0008/433736/004197.pdf

