# Supplementary material for: Allied Health Professional Support in Pediatric Inflammatory Bowel Disease: A Survey from the Canadian Children Inflammatory Bowel Disease Network—A Joint Partnership of CIHR and the CH.I.L.D. Foundation
Source: Can J Gastroenterol Hepatol. 2017 May 16;2017:3676474. doi: 10.1155/2017/3676474 (PMC5448041; doi:10.1155/2017/3676474)
Supplement: Supplementary file 1 — The Supplementary Material displays the questions included in the self-administered questionnaire. [file 3676474.f1.doc]

1. Center’s name:
2. What is the current total number (as accurate as possible) of patients with IBD in your center?
3. Out of the total population (GI, liver and nutrition) seen in your center, what is the average percentage of patients seen in your center with IBD ?
4. What is average total number (as accurate as possible) of newly diagnosed patients with IBD per year?
5. What is the average wait time in weeks for a clinic appointment for new (highly suspicious of) IBD?
6. What is the average wait time in weeks for a clinic appointment for other NEW GI referrals?
7. What is the total number of CLINICAL FTE Pediatric GI physicians in your center?
8. What is the number of FTE CLINICAL Pediatric GI physicians seeing patients with IBD in your center?
9. What is the total number of FTE Pediatric GI nurses or physician assistants in your center?
10. What is the number of FTE Pediatric GI nurses or physician assistants dedicated for patients with IBD in your center?
11. What is the total number of FTE Pediatric GI dieticians in your center/division?
12. What is the number of FTE Pediatric GI dieticians dedicated for patients with IBD in your center/division?
13. How long (in weeks) is the wait time for a new patient with IBD to see a dietician?

WEEKS

1. What is the total number of EFT Pediatric GI social workers in your center/division?
2. What is the number of FTE Pediatric GI social workers dedicated for patients with IBD in your center/division?
3. How long (in weeks) is the wait time for a new patient with IBD to see a social worker?

WEEKS

1. What is the total number of FTE Pediatric clinical psychologists in your center/division?
2. What is the number of FTE Pediatric clinical psychologists dedicated for patients with IBD in your center/division?
3. How long (in weeks) is the wait time for a new IBD patient with to see a clinical psychologist?

WEEKS

1. What is the average wait time (in weeks) for scoping newly diagnosed (highly suspicious of) IBD in your center?

WEEKS

1. What is the average wait time (in weeks) for receiving pathology results for newly diagnosed IBD in your center?

WEEKS

1. What is the average wait time (in weeks) for MRE imaging newly diagnosed IBD in your center?

WEEKS

1. In you center, what’s the average time period (in weeks) between the diagnosis and the first FOLLOW-UP clinic visit?

WEEKS

1. For patients in remission in your center, what is the average time interval (in MONTHS) between routine follow up clinic visits?

MONTHS
